# Sphenopalatine Ganglion Block Using the Tx360 Nasal Applicator for Trigeminal Neuralgia: A Prospective Observational Pilot Study

**DOI:** 10.7759/cureus.78727

**Published:** 2025-02-08

**Authors:** Christina Orfanou, Adamantia Gkaniou, Martina Rekatsina, Aliki Tympa Grigoriadou, Aikaterini Melemeni, Maria Kouri, Abraham Pouliakis, Athanasia Tsaroucha

**Affiliations:** 1 1st Department of Anesthesiology and Pain Medicine, Aretaieion University Hospital, National and Kapodistrian University of Athens, Athens, GRC; 2 Department of Oral Medicine and Oral Pathology and Hospital Dentistry, School of Dentistry, National and Kapodistrian University of Athens, Athens, GRC; 3 2nd Department of Pathology, Attikon University Hospital, National and Kapodistrian University of Athens, Athens, GRC

**Keywords:** neuropathic pain, oro-facial pain, sf-36, sphenopalatine ganglion block, trigeminal neuralgia

## Abstract

Introduction

Trigeminal neuralgia (TN) is a chronic condition characterized by sudden, short episodes of excruciating facial pain affecting one or more branches of the trigeminal nerve (V), which severely impacts patients’ quality of life. Despite the availability of various treatment options, some cases experience poor pain control. Sphenopalatine ganglion (SPG) block using the Tx360 nasal applicator has recently been introduced with promising results as a treatment option in such cases.

Materials and methods

Fifteen adult patients of both sexes suffering from classical or atypical TN involving the maxillary (V2) or mandibular (V3) branches, either partially or completely drug-resistant, with a Numerical Rating Scale (NRS) score of 8-9 despite drug treatment, were enrolled in the study. For six weeks, every seven days, they underwent bilateral SPG block with the Tx360 device (Tian Medical Inc., Lombard, Illinois), receiving 0.3 cc of 2% lidocaine in each block. During the subsequent twelve weeks, patients were followed up monthly, and data regarding TN-related pain symptoms were collected using the verbal rating scale and the Short Form-36 (SF-36) Quality of Life Questionnaire.

Results

All participants exhibited a continuous and significant decrease in NRS scores at each visit during the first six weeks and up to one month after the final SPG block. Subsequently, NRS scores remained stable over the following two months. Significant improvements were noted in all patients in physical functioning, role limitations due to physical health, energy/fatigue, emotional well-being, pain, and general health, as assessed through the SF-36.

Discussion

Our results align with studies of a similar design demonstrating favorable effects of SPG block in various types of head and neck pain, including TN.

Conclusion

Repetitive SPG block with 2% lidocaine administered via the Tx360 device resulted in clinical improvement, significantly reducing NRS scores for a three-month period in patients with TN when used as an add-on to standard treatment. This treatment technique was simple, effective, and not associated with significant adverse effects.

## Introduction

Trigeminal neuralgia (TN) is a chronic condition characterized by sudden, short bursts of acute facial pain affecting one or more branches of the trigeminal nerve (V), significantly impacting the quality of life for those affected [1]. Despite being a rare disease, affecting approximately 4-13 per 100,000 people per year, TN is regarded as the most common form of craniofacial neuropathic pain and is considered one of the most excruciating pain conditions [1]. According to the International Classification of Orofacial Pain, TN is characterized by repeated severe episodes of acute, electric shock-like pain, usually paroxysmal, unilateral, of short duration, and triggered by innocuous stimuli [2].

The causes and underlying mechanisms of TN are poorly understood. All three categories of TN (idiopathic, classical, and secondary) and both disease phenotypes (purely paroxysmal disease or TN accompanied by concomitant continuous pain) are extremely painful, while both diagnosis and treatment can be challenging [2,3].

The initial treatment for TN is pharmacological, usually beginning with a single agent such as carbamazepine or oxcarbazepine. When monotherapy proves ineffective, a combination of different drugs may be administered, including lamotrigine, gabapentin, botulinum toxin type A, pregabalin, baclofen, topiramate, and phenytoin, while intravenous fosphenytoin or lidocaine can be used for acute exacerbation management [1,3]. For patients who do not respond to medication or experience severe side effects, more invasive options such as nerve blocks or surgery may be considered [1].

Sphenopalatine ganglion (SPG) block is an emerging treatment for TN, with relevant studies showing promising results [4,5]. To date, this regional anesthetic technique has also been used with relative success for the treatment of various types of headaches, postherpetic neuralgia, chronic head and neck pain (cancer-related or not), and for providing postoperative analgesia after oral procedures [6-9].

SPG block can be performed through various techniques and approaches, each with its own challenges and risks [4]. The Tx360® nasal applicator (Tian Medical Inc., Lombard, Illinois) has recently been introduced for transnasal SPG block, demonstrating promising results when used repetitively in patients with head and neck pain, including those with TN [7,8,10,11]. The Tx360 device is described as an easy-to-use, cost-effective option that can be quickly implemented, even in emergency settings [8]. It offers precise local anesthetic delivery without the need for a needle and with minimal associated side effects [7,10].

We conducted a prospective observational study using the Tx360 nasal applicator to perform bilateral SPG blocks for the treatment of neuropathic pain in patients with TN. The primary aim was to assess the effectiveness of the SPG block with the Tx360 device as an add-on to the standard treatment that patients were already receiving for their condition. We also evaluated the impact of this treatment on patients' quality of life using the 11-point Numerical Rating Scale (NRS) for pain intensity and the Short Form-36 (SF-36) Quality of Life Questionnaire. The results of this study were previously presented as an oral presentation at the 24th Panhellenic Congress of Regional Anaesthesia, Pain Management & Palliative Care, held on September 19-22, 2024, on Lefkada Island, Greece.

## Materials and methods

This prospective observational study was conducted at Aretaieio University Hospital, Athens, Greece, after obtaining ethical approval from the respective Institutional Review Board (Protocol ID: 274/12-11-2020, Chairman Prof. Konstantoulakis). The study was registered on ClinicalTrials.gov (ID: NCT06663410) and was performed in accordance with the ethical standards outlined in the 1964 Declaration of Helsinki and its later amendments, following the Strengthening the Reporting of Observational Studies in Epidemiology (STROBE) guidelines. The study was conducted from February 2021 to April 2024, including the completion of the follow-up period for the last enrolled patient.

The sample size of 15 participants was selected as this was a pilot trial, and this number was deemed sufficient to provide preliminary insights and evaluate the feasibility of the study design [12]. Twenty-one patients of both sexes, older than 18 years, classified as American Society of Anesthesiologists (ASA) Physical Status I or II, who presented to the Pain Department with TN of any etiology, were assessed for eligibility to participate in the study. Exclusion criteria included contraindications to the use of the Tx360 transnasal applicator (see Table 1), a medical history of allergic reaction to lidocaine, comorbidities with other painful syndromes for which analgesic treatment or the diseases themselves might affect study outcomes, participation in another relevant research study within the last 30 days, pregnancy or breastfeeding, absence of contact details, inability to communicate adequately in Greek or English, and refusal to participate in the study. Written informed consent was obtained from each eligible patient.

**Table 1:** Contraindications to the use of the Tx360® transnasal applicator (Tian Medical Inc., Lombard, Illinois). Data from ref. [10].

List of contraindications to the use of the Tx360 transnasal applicator
Bleeding disorder, coagulation disorders, or any known disease related to the coagulation mechanism
Anatomical deformities of the nasal septum, such as facial or nasal deformities such as cleft lip, cleft palate, choroidal atresia, atrophic rhinitis, medicated rhinitis, perforation of the nasal septum, etc.
Recent surgery of the nasal cavity or paranasal sinuses (<3 months)
Dryness of nasal mucosa, pain in the area, nasal discharge, nosebleeds
Severe respiratory distress of the patient, i.e., on tachypnoea or use of accessory respiratory muscles
Neoplastic diseases such as the following: angiofibroma (juvenile nasopharyngeal angioma), paranasal sinus tumors, granuloma
Nasal congestion of more than 10 days duration, concomitant with high fever, abnormal color of nasal mucous membrane or facial pain or headache
Recent fracture of the nose or skull bones (<3 months)

During the first visit to the clinic, a full medical history was obtained, along with a comprehensive review of TN, including a physical examination, details about diagnosis, disease course, pain characteristics, and past therapeutic interventions (both pharmaceutical and non-pharmaceutical) along with their outcomes. In addition, patients completed the SF-36 Quality of Life Questionnaire to assess the impact of TN pain on their daily activities.

**Figure 1:**
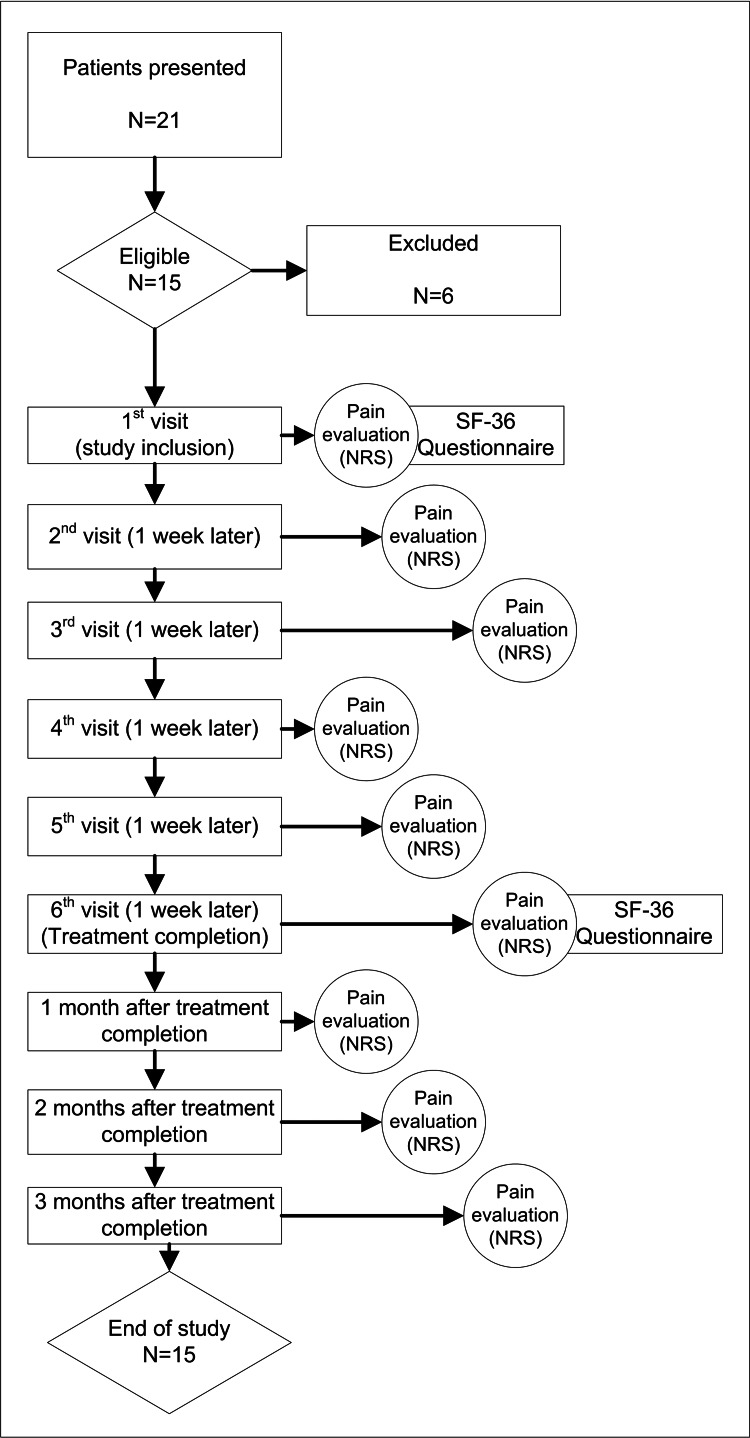
Study flow diagram.

Twenty-one patients presented with either classical or atypical TN affecting the maxillary (V2) or mandibular (V3) branches, partly or completely drug-resistant, with an NRS score of 8-9, under drug treatment, and experiencing minimal or no clinical improvement and/or poor patient satisfaction. Fifteen patients were eligible for study enrollment and were further informed about the study and educated on the implementation of the NRS pain scoring system. Three patients declined to provide signed informed consent, two had significant language barriers, and one was under antithrombotic therapy with poor coagulation monitoring (see flow diagram, Figure 1).

**Figure 2:**
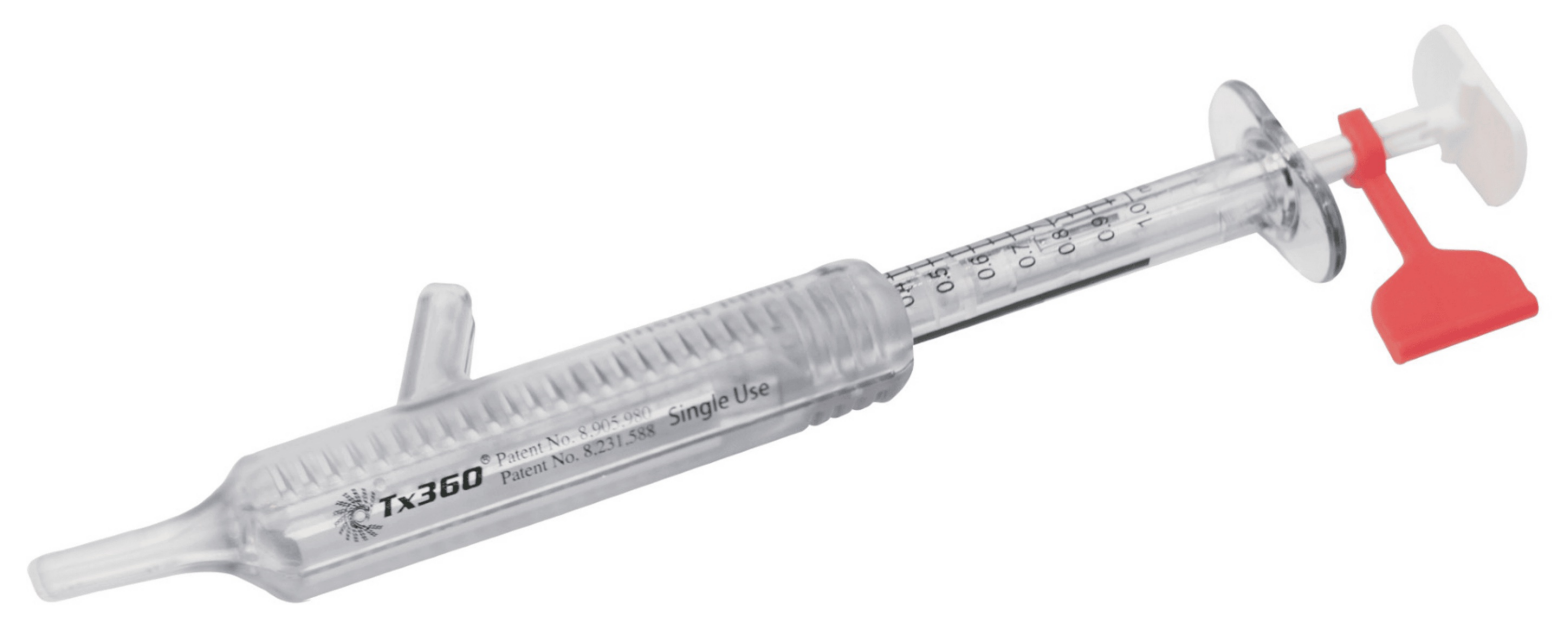
The Tx360® nasal applicator by Tian Medical Inc. (Lombard, Illinois). Image provided by Dr. Tian Xia, DO, Anesthesiologist and manufacturer of the Tx360® nasal applicator (Tian Medical), used with permission.

For each patient, the starting point of study participation was defined as the visit to our pain clinic during which study enrollment was determined. All participants were recommended SPG block using the Tx360 nasal applicator device (see Figure 2) as an add-on therapy to their ongoing standard pharmaceutical treatment for therapy-resistant TN pain symptoms. Most participants had previously undergone several neuropathic pain therapies, including forehead blocks (supraorbital and supratrochlear nerves), trigger point injections, biofeedback, and relaxation techniques, along with different pharmaceutical combinations, with poor pain control and patient satisfaction.

The duration of the study was a total of 18 weeks for each patient. The first six weeks were the treatment period, and the next 12 weeks were the observation period of the participants. The study protocol included six repetitive bilateral transnasal SPG blocks via the Tx360 device, on a strict weekly basis, using the same technique and dosing each time. During each application, a peripheral intravenous catheter was placed to provide adequate venous access in the unlikely event of an anaphylactic reaction to the local anesthetic used. The SPG block was performed using the Tx360 nasal applicator, always by the same experienced, specifically trained anesthesiologist, administering 0.3 cc lidocaine 2% in each nostril.

**Figure 3:**
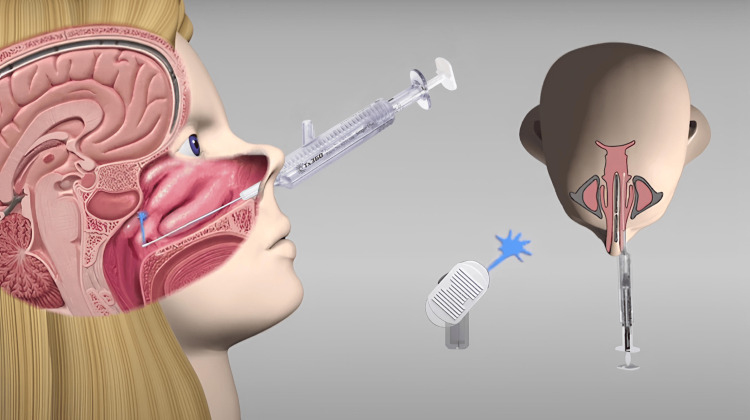
Illustration of sphenopalatine ganglion block using the Tx360® nasal applicator (Tian Medical Inc., Lombard, Illinois). Image provided by Dr. Tian Xia, DO, anesthesiologist and manufacturer of the Tx360 nasal applicator, used with permission.

The SPG block using the Tx360 applicator was conducted in accordance with the manufacturer's instructions, by inserting the tip of the introducer into the affected side’s nostril along the superior aspect of the palatine process of the maxilla (floor of the nose) until the taper transition limited further device advancement. Using the dominant hand, the operator advanced the syringe into the device as the tip of the catheter further protruded out of the introducer until the limit. Upon exiting the introducer, local anesthetic administration followed immediately, and 0.3 cc of the solution was released into the nasal cavity. The operator then retracted the apparatus and repeated the same process on the patient’s other, non-affected side. The whole procedure was considered easy and quick, as a luer-lock syringe of 1 mL volume, pre-loaded with lidocaine, fitted effortlessly into the Tx360 device, and the application resulted in a lidocaine spray upward, laterally, and forward, with a diffusion pattern broad enough to accommodate anatomical variations of the sphenopalatine foramen (see Figure 3). No patient required procedural sedation.

At the end of each procedure, a detailed form was completed, documenting patient data, procedure details, and the occurrence of adverse events (e.g., irritation of the nasal passages, bleeding, anaphylactic reaction). A copy of the form was provided to the patient along with the follow-up schedule.

During the initial six-week treatment period, participants were instructed to record the number of pain episodes/attacks, exacerbation crisis duration, pain intensity (using the NRS), pain characteristics, any precipitating factors, any rescue treatment received, and adverse events related to the current treatment using a thorough pain diary. Upon completion of the treatment period, specifically during the visit when the 6th SPG block was administered (i.e., the 6th visit), patients were instructed to complete the SF-36 questionnaire once again.

During the following 12 weeks, patients were followed up through previously arranged monthly telephone sessions. In these communications, patients were asked about the number of pain episodes/attacks, exacerbation crisis duration, pain intensity (using the NRS), pain characteristics, any precipitating factors, any rescue treatment administration, and the emergence of adverse events related to the study. These data were recorded by the primary investigator in special forms.

Statistical analysis was performed using the programming language R (version 4.4.0, R Foundation for Statistical Computing, Vienna, Austria). Descriptive data are presented as median, minimum, and maximum values, as well as mean and standard deviation (SD). Qualitative data are presented as frequency and relative percentage. Group comparisons for quantitative data were based on the Kruskal-Wallis test, as normality (evaluated by the Shapiro-Wilk test) was not always ensured. For categorical data, the χ² test was applied, with the Fisher exact test used when required. Since patients were evaluated at two time points, paired tests were also applied to determine whether changes in the nine components of the SF-36 questionnaire (physical functioning, role limitations due to physical health, role limitations due to emotional problems, energy/fatigue, emotional well-being, social functioning, pain, general health, perceived health change) were statistically significant. The significance level for the study was set at α < 0.05.

The nine questionnaire components were calculated according to the creator’s instructions (https://www.rand.org/health-care/surveys_tools/mos/36-item-short-form/scoring.html): (a) Categorical values were first substituted with numerical values (from 1 to 6). (b) These values were then converted to a range of 0% to 100%, accounting for the question’s direction. (c) Finally, each component’s value was calculated by averaging the specific questions composing it.

Data collection was closely monitored by an assigned researcher, ensuring the completion of all participant forms with no missing data. Follow-up telephone appointments were carefully tracked and rescheduled in case of unforeseen circumstances preventing their completion, whether on the part of the researcher or the participant.

## Results

Baseline characteristics

This study involved 15 participants with a median age of 58 years (Q1-Q3: 56-67.5, minimum: 50, maximum: 86). Seven participants were male (46.7%). Marital status was summarized as follows: unmarried, 1 (6.7%); divorced, 5 (33.3%); married, 6 (40.0%); and widowed, 3 (20.0%). Five individuals lived alone (33.3%). Participants’ occupational status was summarized as follows: unemployed, 1 (6.7%); civil servant, 2 (13.3%); private employee, 6 (40.0%); domestic worker, 4 (26.7%); and retired, 2 (13.3%).

Participants' characteristics in relation to the questionnaire responses at the 1st and 6th visits are presented in Table 2.

**Table 2:** NRS score at the study time points. The score is presented as a median value with minimum and maximum observation and as a mean value ± standard deviation (SD). NRS: Numerical Rating Scale.

NRS score at the study time points	Median (Min, Max)	Mean±SD
1^st^ visit	8 (7, 9)	8.2±0.78
2^nd^ visit	7 (0, 9)	6.7±2.19
3^rd^ visit	6 (0, 9)	5.2±2.91
4^th^ visit	3 (0, 9)	3.8±3.08
5^th^ visit	3 (0, 9)	3.9±3.04
6^th^ visit	2 (0, 9)	3.4±3
One month after treatment completion	2 (0, 9)	3.1±3.07
Two months after treatment completion	2 (0, 9)	3.1±3.13
Three months after treatment completion	2 (0, 9)	3.1±3.13

Pain evolution over time

**Figure 4:**
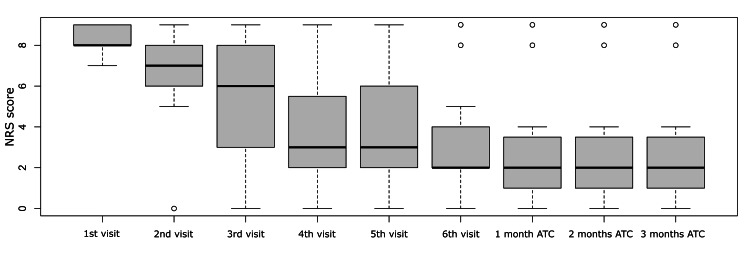
Box-and-whisker plot of NRS scores at various study time points. ATC: after treatment completion, NRS: Numerical Rating Scale.

Figure 4 graphically presents the results of the score analysis for the various study time periods. Notably, there was a continuous decrease in the score at each visit up to one month after treatment. Subsequently, in the second and third months after treatment, the score remained relatively stable. As depicted in Figure 4, two patients (represented by the two outlier dots from the 6th visit onward) exhibited no reduction in their scores throughout the study. Specifically, one patient had a score of 8 and the second had a score of 9. The first patient was a 54-year-old male, and the second was a 57-year-old female, both at the lower end of the study population's age range.

**Figure 5:**
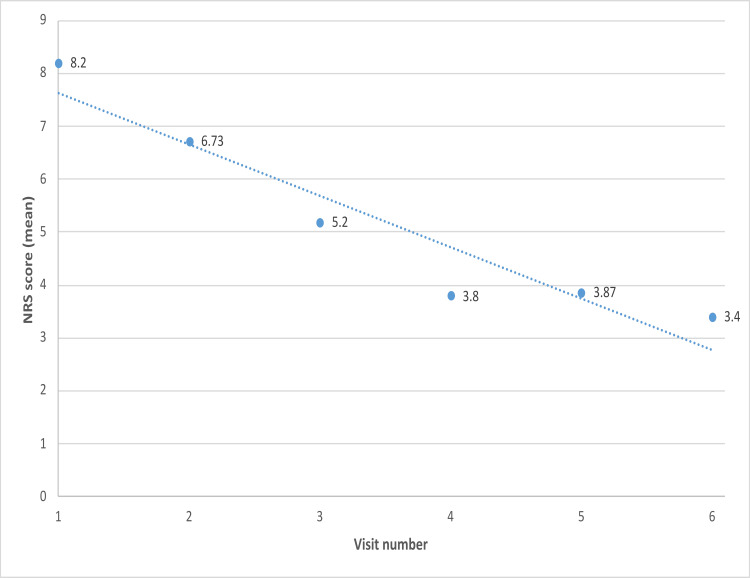
NRS score (mean value) for each one of the study weeks (1–6) along with a fitted line. NRS: Numerical Rating Scale.

To evaluate the rate of pain reduction, as assessed and reported via the NRS score, a significant reduction was observed in the first two months. A linear model was fitted to the data (see Figure 5). The model equation was NRS score = -0.97 × (week number) + 8.6. The model fit was nearly perfect, with a coefficient of determination of R² = 90.1%. This finding indicates that for each week, the NRS score decreased by approximately 1 unit.

Health status evolution as measured by the SF-36 questionnaire

In addition, the difference in the nine components measured by the SF-36 questionnaire at the first visit and at follow-up (6th visit) was evaluated using paired tests, and the results are summarized in Table 3. A higher score is associated with a better health state (i.e., a higher score indicates a more favorable health state compared to a lower score) for all components, as the score is adjusted according to the direction of the questions. Notably, all nine components showed an increase in score from the first visit to follow-up, indicating an overall improvement in health state. Statistically significant differences for the 15 study patients were observed in the following components: physical functioning improved by 9.3 units (20%, p = 0.0121), role limitations due to physical health improved by 43.4 units (326%, p = 0.0054), energy/fatigue improved by 5 units (12%, p = 0.0379), emotional well-being improved by 4.5 units (10.4%, p = 0.0257), pain improved by 23.9 units (70.7%, p = 0.0373), and general health improved by 4 units (11.8%, p = 0.0373). The improvements in components that were not statistically significant were as follows: role limitations due to emotional problems improved by 20 units (64.3%, marginally significant, p = 0.0975), social functioning improved by 3.1 units (6%), and perceived health change improved by 3.3 units (10.4%).

**Table 3:** The nine components measured using the SF-36 questionnaire at the first visit and at the follow-up visit. Data are presented as the mean value along with the standard deviation (SD). The Wilcoxon signed-rank test with continuity correction was performed for all cases, with the significance level set at p < 0.05. W: Wilcoxon W measure.

SF-36 component	First visit (mean ± SD)	Sixth visit (follow-up) (mean ± SD)	p	W
Physical functioning	46.0±32.5	55.3±32.2	0.0121	7
Role limitations due to physical health	13.3±28.1	56.7±46.7	0.0054	0
Role limitations due to emotional problems	31.1±46.2	51.1±50.2	0.0975	0
Energy/fatigue	41.7±18.8	46.7±17.3	0.0379	9.5
Emotional well-being	43.2±18.3	47.7±13.8	0.0257	10.5
Social functioning	51.7±20.5	54.8±25.3	0.2223	9
Pain	33.8±22.4	57.7±30.4	0.0050	5
General health	34.0±22.0	38.0±20.5	0.0373	3
Perceived health change	31.7±17.6	35.0±15.8	0.4237	2.5

## Discussion

Our findings indicate that SPG blockade with 0.3 cc of 2% lidocaine delivered by the Tx360 nasal applicator is beneficial as an add-on to standard treatment for TN. Over the six-week injection cycle, there was a statistically significant reduction in the impact of TN-related pain on patients' daily activities, while satisfaction with analgesia was noted, as assessed by NRS scores.

In our study, we selected 2% lidocaine as the local anesthetic of choice due to its rapid onset of action, effective analgesic properties, and favorable safety profile. Additionally, we opted not to use adjuncts such as dexamethasone or dexmedetomidine to maintain a standardized approach and avoid confounding variables. This ensured that potential favorable results could be attributed directly to lidocaine while avoiding possible adverse effects associated with corticosteroids or α2-adrenergic agonists. Regarding treatment duration and frequency, the optimal therapeutic regimen remains unclear [13]. While a protocol consisting of SPG blocks twice weekly for six weeks has shown promising results in headache treatment [13], we adopted a six-week cycle with one session per week due to financial constraints and to enhance patient adherence. A weekly schedule was deemed more feasible for participants, reducing the burden of frequent clinic visits while maintaining the therapeutic benefits of SPG blockade.

Head and facial pain affects millions of individuals globally, leading to debilitating consequences and costing billions of dollars annually. The impact of these conditions on individuals, families, employers, healthcare systems, and society is considerable [14]. Conditions such as TN impose significant burdens, both personal and societal [15]. Therefore, there is an urgent need for new, effective, and safe therapeutic methods, as conventional treatments appear insufficient for some patients.

Since first being described by Dr. Greenfield Sluder in 1908-1909, when he successfully aborted symptoms of cluster headache with intranasal application of varying concentrations of cocaine, SPG block has been sporadically used to manage head and neck-related pain [15]. In recent years, SPG blockade has been described as an emerging treatment for TN. To date, SPG block has been applied with relative success for headaches, and recent reports describe its use in treating continuous migraine [6], head and neck pain, postoperative analgesia following oral procedures [9], relief of cancer pain in the head and neck area, and postherpetic neuralgia, among other conditions [7,8].

There are several techniques for blocking the SPG, with the most popular being the use of the following: cotton-tip applicator, Tx360 device, nasal spray, and needle injection. The three main approaches are transnasal, transoral, and infrazygomatic. The first three techniques can only be applied via the transnasal route, while needle injection can be performed using all three approaches. Although a variety of local anesthetics have been used, the most commonly used are lidocaine and bupivacaine. The side effects of this block are usually local in nature, including numbness or tingling at the base of the nose and palate, numbness or tearing of the corresponding eye, bitter taste, and numbness in the oral cavity. In techniques involving needle injections, there is a risk of bleeding, infection, and epistaxis [4]. The transnasal approach has several advantages, including technical simplicity, short procedure time, and low overall risks, which are principally limited to epistaxis and infection [5,16]. The advantages of using the Tx360 nasal applicator include precise administration of the local anesthetic without the use of a needle, ease of use, and a low incidence of adverse effects [7,10]. Additionally, this device is economical, portable, and quick to implement, making it suitable even for emergency settings [8].

Although few, relevant studies have shown promising results. The evidence supporting SPG nerve block for most pain syndromes remains at the case report and case series level [4,5].

To the best of our knowledge, this is the first study to examine SPG block using the Tx360 nasal applicator for the treatment of TN in a group of patients. Our results align with studies of a similar setup, demonstrating the favorable effects of SPG block in various types of head and neck pain [7,9,10,11].

A previous randomized controlled trial with 25 participants with refractory second-division TN was conducted by Kanai et al. The patients had been suffering from painful paroxysms for at least three months, with a VAS score greater than 4. The patients were randomized to receive two sprays (0.2 mL) of either 8% lidocaine or saline placebo in the affected nostril using a metered-dose spray. Thirteen patients received lidocaine first, while twelve received placebo first. Paroxysmal pain triggered by touching or moving the face was assessed using the VAS score before and 15 minutes after application. Thirty minutes post-application, patients were asked to characterize their overall pain response as either stable, progressively improved, or temporarily improved followed by a return of discomfort. Additionally, they were required to maintain a pain diary to log their pain levels and provide a detailed description of their experience at their scheduled follow-up appointment seven days post-therapy. The intranasal 8% lidocaine spray led to a substantial reduction in the VAS score, dropping from an average of 8.0 before administration to 1.5 at 15 minutes after use. In contrast, the placebo spray showed no significant change in the VAS score. Results suggested that intranasal 8% lidocaine spray significantly decreased paroxysmal pain for an average of 4.3 hours. The side effects were limited to local discomfort, including burning, stinging, or numbness of the nose and eye, along with a bitter taste and numbness of the throat [17].

In another study, four patients with refractory TN were treated with a single transnasal SPG block using the Sphenocath device, injecting 3 mL of 2% lidocaine on the affected side. The 11-point Numeric Rating Scale (NRS) for pain was measured before and 15 minutes after treatment. The Patient Global Impression of Change (PGIC) was recorded at both the 15-minute mark and the two-week follow-up, with ratings categorized as very good, good, no change, poor, or very poor. Additionally, the Brief Pain Inventory - Facial (BPI-F), which assesses pain intensity, general activity interference, and facial-specific pain interference, was evaluated before and two weeks post-treatment. All four patients reported significant pain relief at 15 minutes post-injection, with the average baseline NRS score decreasing from 9 to 0.75. After 14 days, there was improvement in the BPI-F score, showing pain intensity reduced by 60%, interference with daily activities by 82%, and facial-specific pain interference by 61%. Each patient rated their outcomes as either good or very good on the PGIC scale. Reported adverse effects were mild, including throat numbness, nasal discomfort, dizziness, and nausea [18].

It is worth noting that in the aforementioned studies, the analgesic effect of the treatment was evaluated within minutes and days, in contrast to our study, where a prolonged effect was assessed [17,18]. Despite the small number of subjects in our study, there was no loss to follow-up at three months post-treatment, allowing us to obtain valuable information about the long-term effects of repetitive SPG blockade with 0.3 cc of 2% lidocaine via the Tx360 device.

One case series investigated the use of a combination of dexamethasone and ropivacaine with the Tx360 applicator, which resulted in short-term pain relief in the treatment of trigeminal neuralgia, chronic migraine, and postherpetic neuralgia in three female patients (ages 43, 18, and 15). All patients reported significant pain relief within 15 to 30 minutes post-treatment. Two of the three patients experienced consistently low pain levels during follow-up, though a slight reduction in pain relief was observed by days 21 and 28. The third patient initially experienced significant pain relief, but by the day 7 follow-up, pain levels returned to baseline. Patients underwent up to 10 SPG blocks over one year as needed, following the initial 28-day period, with no serious adverse effects, except for one case of mild transient nasal bleeding. These findings suggest that adjuvants such as dexamethasone may enhance SPG blockade, but further studies are required to confirm their long-term efficacy [8].

This previous study showed that performing multiple blocks repeatedly over time could potentially disrupt the pain cycle more effectively by providing a more sustained modulation of autonomic pathways. However, determining the long-term efficacy remains challenging in the absence of further studies.

Limitations

This study has some limitations. Firstly, our study population consisted of only 15 patients due to several factors, the most significant being the low incidence of the examined disease and the limited capacity of our pain clinic, which is constrained by both material and human resource shortages. Secondly, our study did not include a placebo group, as the condition under investigation causes excruciating pain, and the research team deemed it unethical to withhold analgesia from any group. Additionally, patients covered the cost of the special nasal applicator used for the procedure, making it inappropriate to ask anyone to pay for a placebo and potentially not receive any therapeutic benefit. Thirdly, and directly related to the preceding limitation, funding shortages restricted the treatment period to six weeks, with one SPG block administered bilaterally per week. Furthermore, the follow-up period was intentionally limited to three months to minimize the risk of patient attrition during this study phase. A longer follow-up period may have provided greater insight into the gradual reduction of initial therapeutic benefits over time.

## Conclusions

This study showed that repetitive SPG block with 0.3 cc of 2% lidocaine bilaterally using the Tx360 nasal applicator appears to provide beneficial results as an add-on treatment for drug-resistant TN. Statistically significant pain relief was observed for a three-month period post-treatment. In addition, the Tx360 device was simple to use and was not associated with any statistically significant or lasting adverse events. Further studies, particularly randomized controlled trials with larger patient populations, are needed to investigate the optimal SPG interval, dosing, and frequency of follow-up applications.
